# Arachidonic Acid Added during the Differentiation Phase of 3T3-L1 Cells Exerts Anti-Adipogenic Effect by Reducing the Effects of Pro-Adipogenic Prostaglandins

**DOI:** 10.3390/life13020367

**Published:** 2023-01-29

**Authors:** Michael N. N. Nartey, Mitsuo Jisaka, Pinky Karim Syeda, Kohji Nishimura, Hidehisa Shimizu, Kazushige Yokota

**Affiliations:** 1 The United Graduate School of Agricultural Sciences, Tottori University, 4-101 Koyama-Minami, Tottori 680-8553, Japan; 2 Council for Scientific and Industrial Research-Animal Research Institute, Achimota, Accra P.O. Box AH20, Ghana; 3 Department of Life Science and Biotechnology, Shimane University, 1060 Nishikawatsu-Cho, Shimane, Matsue 690-8504, Japan; 4 Institute of Agricultural and Life Sciences, Academic Assembly, Shimane University, 1060 Nishikawatsu-Cho, Shimane, Matsue 690-8504, Japan; 5 Interdisciplinary Center for Science Research, Shimane University, 1060 Nishikawatsu-Cho, Shimane, Matsue 690-8504, Japan

**Keywords:** arachidonic acid, 6-PUFAs, prostaglandin, the differentiation phase, adipogenesis

## Abstract

A linoleic acid (LA) metabolite arachidonic acid (AA) added to 3T3-L1 cells is reported to suppress adipogenesis. The purpose of the present study aimed to clarify the effects of AA added during the differentiation phase, including adipogenesis, the types of prostaglandins (PG)s produced, and the crosstalk between AA and the PGs produced. Adipogenesis was inhibited by AA added, while LA did not. When AA was added, increased PGE_2_ and PGF_2α_ production, unchanged Δ^12^-PGJ_2_ production, and reduced PGI_2_ production were observed. Since the decreased PGI_2_ production was reflected in decreased CCAAT/enhancer-binding protein-β (C/EBPβ) and C/EBPδ expression, we expected that the coexistence of PGI_2_ with AA would suppress the anti-adipogenic effects of AA. However, the coexistence of PGI_2_ with AA did not attenuate the anti-adipogenic effects of AA. In addition, the results were similar when Δ^12^-PGJ_2_ coexisted with AA. Taken together, these results indicated that the metabolism of ingested LA to AA is necessary to inhibit adipogenesis and that exposure of AA to adipocytes during only the differentiation phase is sufficient. As further mechanisms for suppressing adipogenesis, AA was found not only to increase PGE_2_ and PGF_2α_ and decrease PGI_2_ production but also to abrogate the pro-adipogenic effects of PGI_2_ and Δ^12^-PGJ_2_.

## 1. Introduction

White adipose tissue is a major metabolic organ responsible for energy homeostasis [1]. The status of adipocytes significantly affects the development of obesity and diabetes [2], two typical noncommunicable diseases that have recently become major problems in developed countries. Preadipocytes in the immediate vicinity of adipocytes in white adipose tissue can alter adipose tissue function by differentiating into adipocytes. Established immortal preadipocyte cell lines such as 3T3-L1 cells have facilitated studies of adipogenesis [3,4]. In general, adipogenesis is initiated in vitro by incubating confluent 3T3-L1 preadipocytes in a medium containing a mixture of 3-isobutyl-1-methylxanthine (IBMX), dexamethasone, and insulin (MDI). MDI triggers the post-confluence mitotic clonal expansion of cells at G0/G1 growth arrest, forcing the cells to exit the cell cycle into terminal differentiation. IBMX and dexamethasone rapidly induce the expression of CCAAT/enhancer-binding protein-β (C/EBPβ) and C/EBPδ, respectively, during the differentiation phase. These transcription factors further induce the expression of the key adipogenic transcription factors C/EBPα and peroxisome proliferator-activated receptor-γ (PPARγ) [5,6,7,8,9] that evokes a positive feedback loop between them PPARγ and C/EBPα [10]. Thereafter, the expression of genes prerequisite to realize the adipocyte phenotype is induced [11,12,13].

Arachidonic acid (AA), an n-6 polyunsaturated fatty acid (n-6 PUFA), is biosynthesized from dietary linoleic acid (LA) and is involved in the production of pro- or anti-adipogenic prostaglandins (PG)s via the metabolism initiated by cyclooxygenase (COX) isoforms, COX-1 and COX-2. Pro-adipogenic PGs include PGI_2_ [14,15] and the PGJ_2_ derivatives, 15-deoxy-Δ^12,14^-PGJ_2_ (15d-PGJ_2_) and Δ^12^-PGJ_2_ [16,17,18,19]. PGI_2_ rapidly upregulates C/EBPβ and C/EBPδ expression by activating the IP receptor in preadipocytes [20]. Furthermore, the activation of IP receptors by PGI_2_ is also suggested to activate PPARγ by activating IP receptors in HEK293 cells [21]. In addition, both 15d-PGJ_2_ and Δ^12^-PGJ_2_ are representative PPARγ activators, which lead to adipogenesis [16,17]. Anti-adipogenic PGs include PGE_2_ [22,23,24] and PGF_2α_ [25,26]. PGE_2_ and PGF_2α_ are reported to suppress adipogenesis by activating their G protein-coupled receptors, EP4, and FP receptors, respectively [23,24,25,26].

Over 85% of the total PUFAs consumed in Western diets are n-6 PUFAs, mainly LA, a precursor of AA [27]. The composition of fatty acids in red blood cells from mice fed for 53 days with corn oil that is rich in LA, but without the detectable amount of AA, confirmed that the n-6 PUFAs in the diet were converted to AA [28]. In addition, the effects of n-6 PUFAs on adipose tissue were evaluated based on the balance of dietary carbohydrates and proteins. A higher carbohydrate/protein ratio in the diet results in a higher plasma insulin/glucagon ratio, an environment in which dietary n-6 PUFAs promote adipose tissue expansion [28]. Conversely, a higher protein/carbohydrate ratio in the diet increases the plasma glucagon/insulin ratio and promotes cAMP-dependent signaling. The synthesis of PGE_2_ and PGF_2α_ mediated by COX is enhanced in this environment, and these PGs reduce white adipose tissue mass [28]. The balance of carbohydrates and proteins in the diet affected cAMP levels in adipocytes in the experimental animals.

Because IBMX is a cAMP-elevating agent, incubation of 3T3-L1 cells with this compound during the differentiation phase may mimic the high protein/carbohydrate ratio in the diet. Indeed, adipogenesis is promoted when 3T3-L1 cells are incubated during the differentiation phase with AA, dexamethasone, and insulin, except IBMX [28,29,30]. The biosynthesis of PGE_2_ and PGF_2α_ is decreased, whereas that of PGI_2_ is increased under these culture conditions [30]. In contrast, adding MDI and AA during the differentiation phase of 3T3-L1 cells increases the biosynthesis of PGE_2_ and PGF_2α_ [28,31], and suppresses MDI-induced adipogenesis [28,29,31]. However, because there are no reports on which prostaglandins other than PGE_2_ and PGF_2α_ are produced by AA added during the differentiation phase, nor on how the crosstalk between PGs produced and AA during the differentiation phase affects adipogenesis, the present study thus aimed to analyze these points. Furthermore, since LA has pro-adipogenic effects in the absence of IBMX during the differentiation phase of 3T3-L1 cells [30], we examined the effect of LA in the presence of IBMX.

## 2. Materials and Methods

### 2.1. Materials

Dulbecco’s modified Eagle medium containing 25 mM HEPES (DMEM-HEPES), penicillin G potassium salt, streptomycin sulfate, dexamethasone, fatty acid-free bovine serum albumin, and recombinant human insulin was obtained from Sigma-Aldrich Co. (St. Louis, MO, USA). L-Ascorbic acid phosphate magnesium salt n-hydrate, and 3-isobutyl-1-methylxanthine (IBMX) were from Wako Pure Chemical Industries Ltd. (Osaka, Japan). Fetal bovine serum (FBS) was purchased from MP Biomedicals (Solon, OH, USA). AA, LA, PGE_2_, PGF_2α_, Δ^12^-PGJ_2_, and 6-keto-PGF_1α_ were from Cayman Chemical (Ann Arbor, MI, USA). We purchased M-MLV reverse transcriptase (point mutation without Ribonuclease H activity) from Promega (Madison, WI, USA). Oligonucleotides for real-time quantitative (RT-q) PCR amplification were provided by Sigma Genosys Japan (Ishikari, Japan).

### 2.2. Culture of 3T3-L1 Cells and Induction of Adipogenesis 

Mouse 3T3-L1 pre-adipogenic cells (JCRB9014; JCRB Cell Bank, Osaka, Japan) in the growth phase were seeded at a density of 1 × 10^5^ or 2 × 10^5^ in 35 or 60 mm dishes containing 2 or 4 mL, respectively, of growth medium (GM; DMEM-HEPES supplemented with 10% FBS, penicillin G [100 units/mL], streptomycin sulfate [100 μg/L], and ascorbic acid [200 μM]), then incubated at 37 °C under 7% CO_2_ until they reached confluence. Confluent monolayers were incubated with differentiation medium (DM; GM supplemented with dexamethasone [1 μM], IBMX [0.5 mM], and insulin [10 μg/mL]) for 6–48 h to induce differentiation into adipocytes. The cells were then incubated for 6–10 days in maturation medium (MM; GM supplemented with insulin [5 μg/mL]). The medium was replaced with fresh MM every 2 days to promote the accumulation of fat in 3T3-L1 cells during maturation. We examined the effects of various agents added during differentiation on adipogenesis by incubating confluent cell monolayers in DM supplemented with test compounds for 6–48 h, followed by the standard maturation protocol. The test compounds were dissolved in ethanol and added to the DM to a final ethanol concentration of 0.2%.

### 2.3. Quantification of Intracellular TAGs and Cellular Proteins

Cultured mature adipocytes were harvested, suspended in phosphate-buffered saline (PBS) without Ca^2+^ and Mg^2+^ (PBS [–]) supplemented with 0.05% trypsin and 0.53 mM EDTA, and incubated at 37 °C for 5 min. The resulting cell suspensions were washed with PBS (-), divided into two portions, and homogenized in 25 mM Tris-HCl buffer (pH 7.4) containing 1 mM EDTA and 1 N NaOH. Amounts of intracellular TAGs were quantified in one portion using Triglyceride E-Test Kits (Wako). Cellular proteins were precipitated in the other portion with ice-chilled 6% trichloroacetic acid to remove interfering substances and then quantified using the Lowry method with fatty acid-free bovine serum albumin as a standard. Amounts of intracellular TAGs were normalized to the protein content and are expressed as relative amounts of accumulated intracellular TAGs.

### 2.4. Quantification of PGs by ELISA

Levels of PGE_2_, PGF_2α_, Δ^12^-PGJ_2_, and 6-keto-PGF_1α_ were measured in the DM containing IBMX in the presence 50 μM AA for 48 h. We quantified PGE_2_ by ELISA specific for PGE_2_ as described [32]. A PGE_2_-conjugate and fatty acid-free bovine serum albumin were immobilized in 96-well microplates. Immobilized antigen in standards or test samples was competitively incubated with diluted mouse monoclonal antibody specific for PGE_2_. The resultant immunocomplex was detected spectrophotometrically by monitoring the peroxidase activity using o-phenylenediamine as a substrate after binding to biotin-conjugated rabbit anti-mouse IgG antibody and ExtrAvidin peroxidase conjugate as described [33]. We determined the amounts of Δ^12^-PGJ_2_ using monoclonal antibodies specific for Δ^12^-PGJ_2_. Polyclonal mouse antisera specific for PGF_2α_ and 6-keto-PGF_1α_ that reflect PGI_2_ biosynthesis were used to develop solid-phase ELISA for the corresponding immobilized antigens as described [34]. Standard curves were generated in fresh DM containing IBMX to quantify PGs including PGE_2_, PGF_2α_, Δ^12^-PGJ_2_, and 6-keto-PGF_1α_, which were biosynthesized during differentiation.

### 2.5. Quantification of Gene Expression

Total RNA (1 μg) extracted from the cells after 6, 24, and 48 h of the differentiation phase, and on day 6 of the maturation phase using acid guanidium thiocyanate/phenol/chloroform was reverse transcribed (RT) using M-MLV reverse transcriptase (Point mutation without Ribonuclease H activity). Single-stranded cDNA was synthesized using oligo-(dT)_15_ and a random 9-mer (Promega) as primers in the RT reaction. Transcript levels were determined by RT-qPCR using TB GreenTM Premix Ex TaqTM II (Tli RNaseH Plus) kits (Takara Bio Co., Inc., Kusatsu, Japan) and a Thermal Cycler Dice^TM^ Real Time System (Takara Bio Co., Inc.) according to the threshold cycle (CT) and ^ΔΔ^CT methods described by the manufacturer. Table 1 shows the oligonucleotides used herein. The cycling program comprised 95 °C for 30 s, 40 cycles at 95 °C for 5 s and 60 °C for 30 s, followed by 95 °C for 15 s and 60 °C for 30 s. Levels of target gene transcripts were determined and normalized to those of *β-Actin*. The accession numbers of the target genes are as follows: *C/Ebpβ*, NM_009883; *C/Ebpδ*, NM_007679; *Pparγ*, NM_011146; *C/Ebpα*, NM_001287523; *Lpl*, NM_008509; *Glut4*, AB008453; *Leptin*, NM_008493; *β-Actin*, NM_007393.

### 2.6. Statistical Analyses

All results are expressed as means ± standard error of the mean (SEM). Data were statistically analyzed by Dunnett tests, Student *t*-tests, and Tukey-Kramer tests using Excel 2011 (Microsoft Corp., Redmond, WA, USA) and Statcel 4 (OMS Publishing Co., Saitama, Japan). Values with *p* < 0.05 were considered statistically significant.

## 3. Results

### 3.1. Effects of AA or LA Added during Only the Differentiation Phase of 3T3-L1 Cells on MDI-Induced Adipogenesis

**Figure 1:**
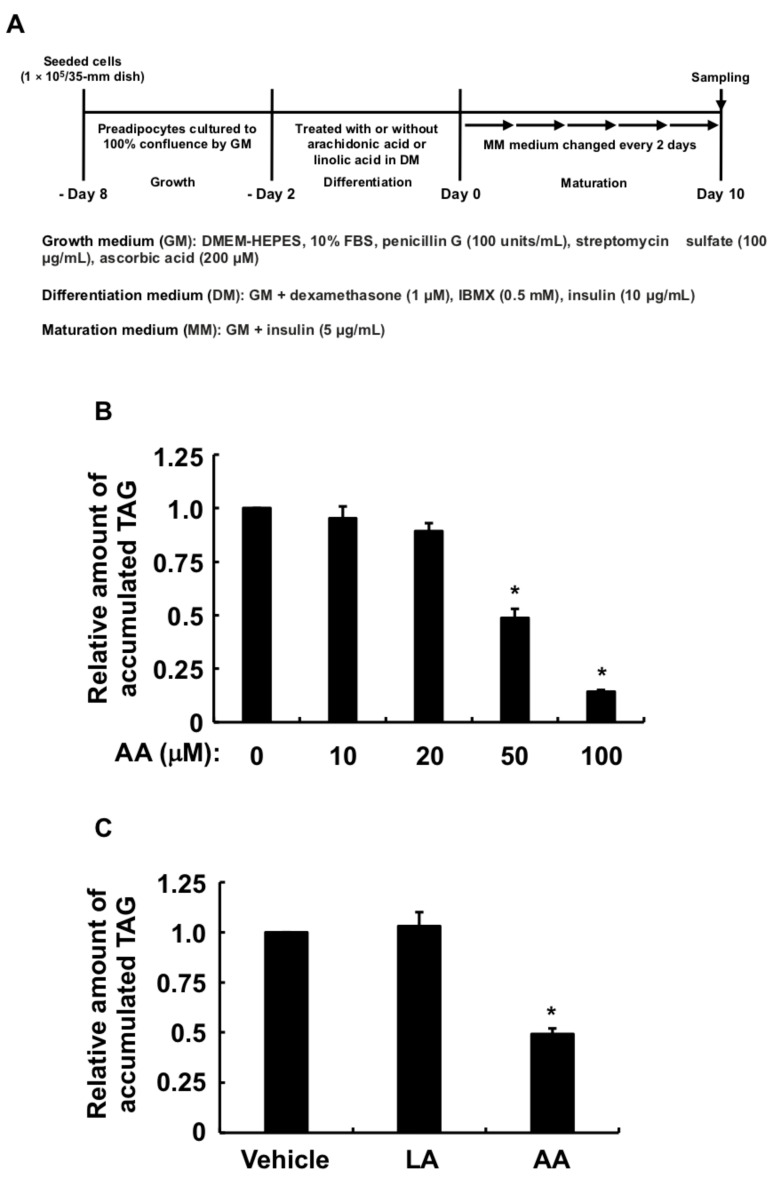
Effects of AA or LA added during the differentiation phase of 3T3-L1 cells on intracellular TAG levels after maturation. (**A**) Experimental procedure. We seeded 3T3-L1 cells (1 × 10^5^/dish) in 35 mm dishes containing 2 mL of GM and incubated them until they reached 100% confluence. Confluent cells were cultured for 48 h in 2 mL of DM-containing vehicle, indicated concentrations of AA, or LA (50 µM), followed by 2 mL of fresh MM every 2 days for 10 days. Intracellular TAG levels were analyzed in terminally differentiated mature adipocytes collected on day 10. (**B**,**C**) Intracellular TAG levels in cultured adipocytes. Data represent means ± SEM of n = 3 experiments for (**B**,**C**). * *p* < 0.05 vs. control (vehicle in DM) (Dunnett tests). GM, growth medium; DM, differentiation medium; MM, maturation medium; AA, arachidonic acid; LA, linoleic acid; TAG, triacylglycerol; SEM, standard error of the mean.

The present study examined whether AA added during the differentiation phase affects the anti-adipogenic effects following the procedure in Figure 1A. As an indicator for adipogenic differentiation of 3T3-L1 cells, we evaluated intracellular TAG accumulation. AA added up to 100 µM during the differentiation phase of 3T3-L1 cells dose-dependently reduced the accumulation of intracellular TAG in MDI-induced mature adipocytes (Figure 1B). Due to the fact that 50 µM AA was sufficient to be effective, we used this concentration in subsequent analyses. We next checked whether LA, like AA, also suppresses MDI-induced adipogenesis following the procedure in Figure 1A because AA in humans is largely produced from LA ingested in the diet. Figure 1C shows that LA, unlike AA, added during the differentiation phase did not inhibit the accumulation of intracellular TAG in MDI-induced mature adipocytes. These results indicated that the metabolic conversion of LA to AA is needed to suppress the accumulation of intracellular TAG during maturation induced by MDI. In addition, if 3T3-L1 cells are exposed to AA during the differentiation phase, its anti-adipogenic effects can be sufficiently exerted even without exposure to AA during the maturation phase.

### 3.2. Types of PGs Biosynthesized by Adding AA during Only the Differentiation Phase of 3T3-L1 Cells and Effects of PGs Added during Only the Differentiation Phase on MDI-Induced Adipogenesis

**Figure 2:**
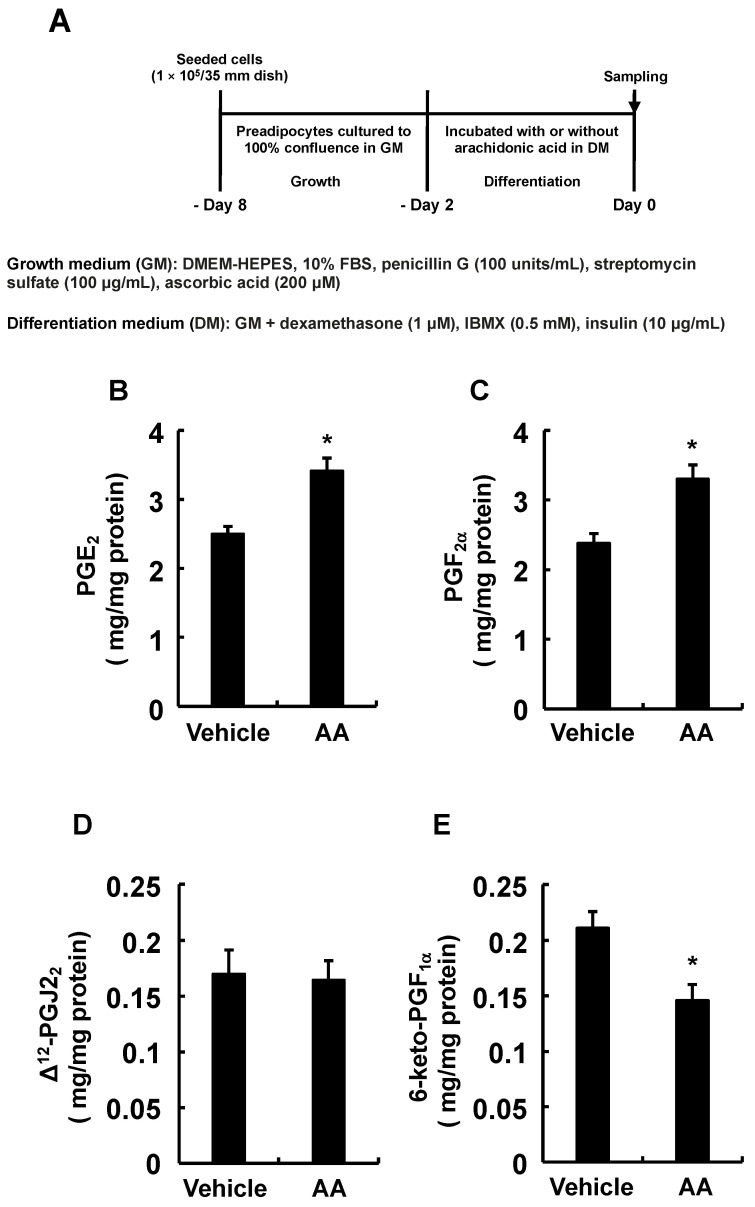
Effect of AA added during the differentiation phase of 3T3-L1 cells on endogenous PG biosynthesis. (**A**) Experimental procedure. We seeded 3T3-L1 cells (1 × 10^5^/dish) in 35 mm dishes containing 2 mL of GM and cultured them until they reached 100% confluence. Confluent cells were cultured for 48 h in 2 mL of DM-containing vehicle or AA (50 µM). The culture media was collected to determine PGs. (**B**) PGE_2_, (**C**) PGF_2α_, (**D**) Δ^12^-PGJ_2_, (**E**) 6-keto-PGF_1α_ assessed by ELISA using specific antibodies. Data are shown as means ± SEM of n = 3 experiments for (**B**–**E**). * *p* < 0.05 vs. control (vehicle in DM) (Student *t*-tests). GM, growth medium; DM, differentiation medium; AA, arachidonic acid; PG, prostaglandin; PGE_2_, prostaglandin E_2_; PGF_2α_, prostaglandin F_2α_; Δ^12^-PGJ_2_, Δ^12^-prostaglandin J_2_; 6-keto-PGF1_α_, 6-keto-prostaglandin F_1α_; SEM, standard error of the mean.

**Figure 3:**
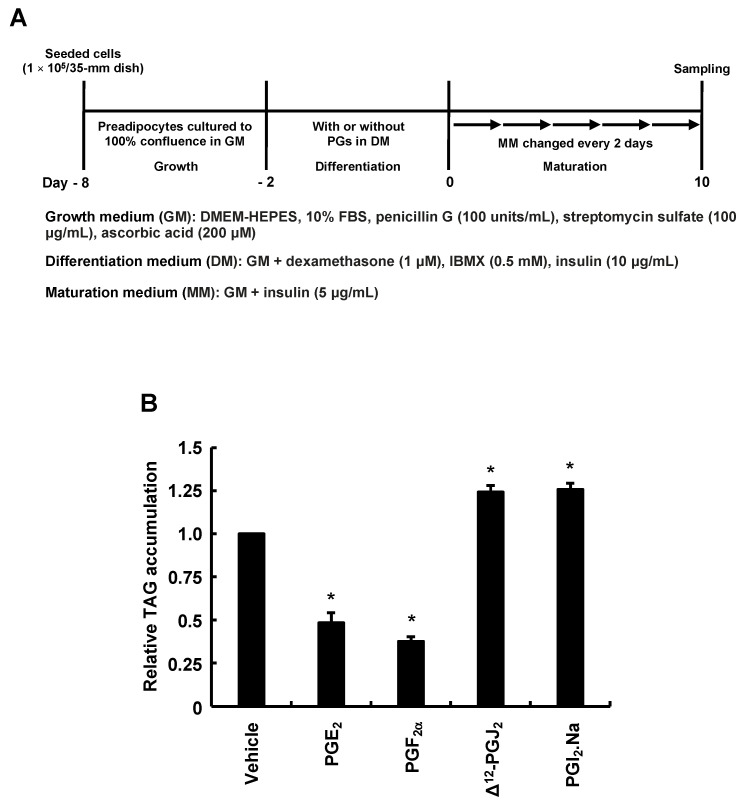
Effects of PGs added during the differentiation phase of 3T3-L1 cells on intracellular TAG levels after maturation. (**A**) Experimental procedure. We seeded 3T3-L1 cells (1 × 10^5^/dish) in 35 mm dishes containing 2 mL of GM and incubated them until they reached 100% confluence. Confluent cells were cultured for 48 h in 2 mL of DM-containing vehicle, PGE_2_ (1 µM), PGF_2α_ (1 µM), Δ^12^-PGJ_2_ (1 µM), or PGI_2_. Na (100 nM), followed by 2 mL of fresh MM every 2 days for 10 days. Intracellular TAG levels were analyzed in terminally differentiated mature adipocytes collected on day 10. (**B**) Intracellular TAG levels in cultured adipocytes. Data represent means ± SEM of n = 3 experiments for (**B**). * *p* < 0.05 vs. control (vehicle in DM) (Dunnett tests). GM, growth medium; DM, differentiation medium; MM, maturation medium; PG, prostaglandin; PGE_2_, prostaglandin E_2_; PGF_2α_, prostaglandin F_2α_; Δ^12^-PGJ_2_, Δ^12^-prostaglandin J_2_; PGI_2_.Na, prostaglandin I_2_.Na; TAG, triacylglycerol; SEM, standard error of the mean.

We evaluated what PGs were produced by adding AA during the differentiation phase. Analysis performed according to the procedure in Figure 2A confirmed the elevated PGE_2_ and PGF_2α_ production, similar to previous reports (Figure 2B,C) [28,31]. Since the production of other PGs is unknown, we checked whether other PGs are also synthesized. The production of Δ^12^-PGJ_2_ was not altered under these conditions (Figure 2D), while that of PGI_2_-derived 6-keto-PGF_1α_ was decreased, suggesting decreased PGI_2_ production (Figure 2E). As PGI_2_ is rapidly degraded to stable 6-keto-PGF_1α_, the amount of 6-keto-PGF_1α_ reflects that of PGI_2_ [34]. Based on the results of Figure 2, we examined how MDI-induced adipogenesis would be affected if 3T3-L1 cells responded to PGs during only the differentiation phase (Figure 3A). PGE_2_ and PGF_2α_ added during the differentiation phase inhibited MDI-induced adipogenesis during the maturation phase (Figure 3B). On the other hand, Δ^12^-PGJ_2_, and PGI_2_ added during the differentiation phase promoted MDI-induced adipogenesis during the maturation phase (Figure 3B). Together with the results in Figure 2, the suppression of MDI-induced adipogenesis during the maturation phase by AA added during the differentiation phase may be due not only to increased anti-adipogenic PGE_2_ and PGF_2α_ but also to reduced pro-adipogenic PGI_2_ production during the differentiation phase.

### 3.3. Adipogenesis-Related Gene Expression in Response to AA during the Differentiation Phase

**Figure 4:**
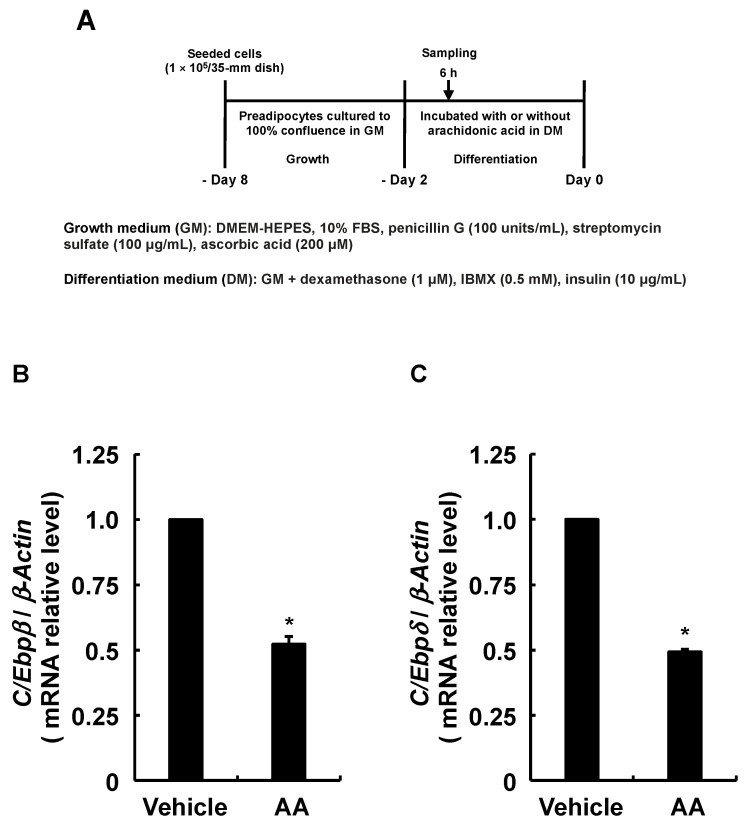
Effects of AA added during the differentiation phase of 3T3-L1 cells on expression of *C/Ebpβ* and *C/Ebpδ*. (**A**) Experimental procedure. We seeded 3T3-L1 cells (2 × 10^5^/dish) in 60 mm dishes containing 4 mL of GM and incubated them until they reached confluence. Thereafter, cells were incubated with DM containing vehicle or AA (50 µM) during the differentiation phase for 6 h. Expression of mRNA for (**B**) *C/Ebpβ* and (**C**) *C/Ebpδ* determined by RT-qPCR. Data are shown as means ± SEM of n = 3 experiments for (**B**,**C**). * *p* < 0.05 vs. control (vehicle in DM) (Student *t*-tests). GM, growth medium; DM, differentiation medium; AA, arachidonic acid; *C/Ebpβ, CCAAT/enhancer-binding protein-β; C/Ebpδ, CCAAT/enhancer-binding protein-δ;* SEM, standard error of the mean.

**Figure 5:**
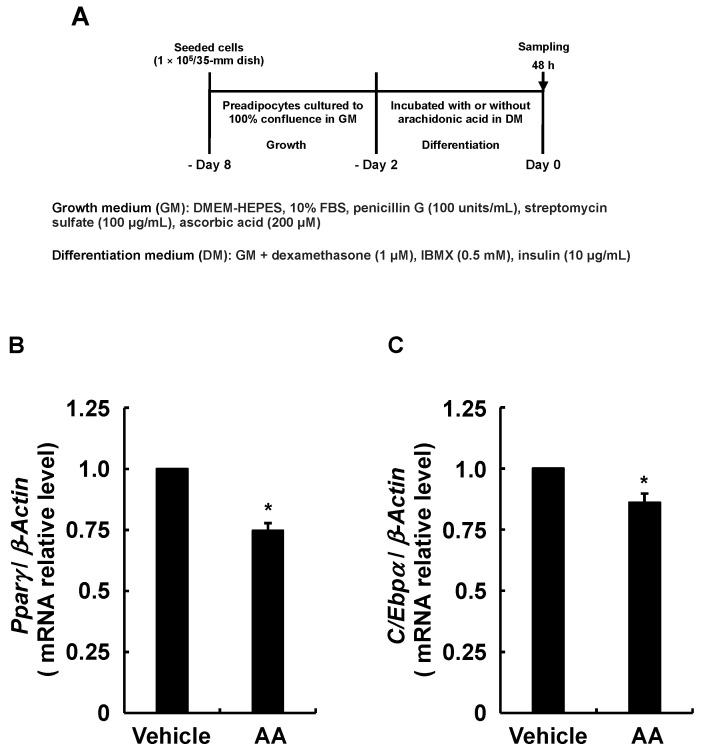
Effects of AA added during the differentiation phase of 3T3-L1 cells on expression of *Pparγ* and *C/Ebpα*. (**A**) Experimental procedure. We seeded 3T3-L1 cells (2 × 10^5^/dish) in 60 mm dishes in 4 mL of GM and incubated them until they reached confluence. Thereafter, cells were incubated with DM containing vehicle or AA (50 µM) during the differentiation phase for 48 h. Expression of mRNA for (**B**) *Pparγ* and (**C**) *C/Ebpα* determined by RT-qPCR. Data are shown as means ± SEM of n = 3 experiments for (**B**,**C**). * *p* < 0.05 vs. control (vehicle in DM) (Student *t*-tests). GM, growth medium; DM, differentiation medium; AA, arachidonic acid; *Pparγ*, *proliferator-activated receptor-γ*; *C/Ebpα*, *CCAAT/enhancer-binding protein-α*; SEM, standard error of the mean.

**Figure 6:**
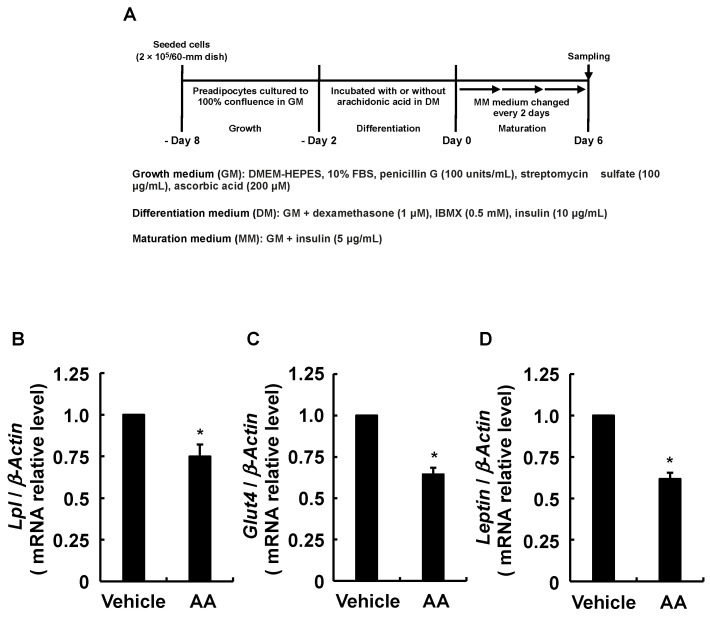
Effects of AA added during the differentiation phase of 3T3-L1 cells on expression of *Lpl*, *Glut4*, and *Leptin*. (**A**) Experimental procedure. We seeded 3T3-L1 cells (2 × 10^5^/dish) in 60 mm dishes containing 4 mL of GM and incubated them until they reached confluence. Thereafter, cells were incubated with DM-containing vehicle or AA (50 µM) during the differentiation phase, followed by 6 days in fresh MM that was replaced every 2 days. Expression of mRNA for (**B**) *Lpl*, (**C**) *Glut4*, and (**D**) *Leptin* determined by RT-qPCR. Data are shown as means ± SEM of n = 3 experiments for (**B**–**D**). * *p* < 0.05 vs. control (vehicle in DM) (Student *t*-tests). GM, growth medium; DM, differentiation medium; MM, maturation medium; AA, arachidonic acid; *Lpl*, *lipoprotein lipase*; *Glut4*, *glucose transporter 4*; SEM, standard error of the mean.

Although endogenous PGI_2_ has a very short chemical lifespan [35], we found that PGI_2_ induces adipogenesis by activating IP receptors in an autocrine fashion before spontaneous degradation [30]. Furthermore, PGI_2_ added during the differentiation phase significantly promoted the adipogenesis of 3T3-L1 cells (Figure 3B). Therefore, since the results in Figure 2E suggested that AA decreased PGI_2_ production, we predicted that induction of the MDI-induced adipogenic program would be suppressed, starting with activated IP receptor to increase *C/Ebpβ* and *C/Ebpδ* expression [20]. The expression of *C/Ebpβ* and *C/Ebpδ* was analyzed as described in Figure 4A. The RT-qPCR findings show that AA added during the differentiation phase reduced the expression of *C/Ebpβ* and *C/Ebpδ* which are critical for the progression of the early phase of adipogenesis (Figure 4B,C). Since *C/Ebpβ* and *C/Ebpδ* levels affect the expression of *Pparγ* and *C/Ebpα*, master regulators of adipogenesis [5,6,7,8,9], we analyzed their expression as described in Figure 5A. The RT-qPCR revealed reduced *Pparγ* and *C/Ebpα* expression, which should result from the downregulation of *C/Ebpβ* and *C/Ebpδ* (Figure 5B,C). Due to the fact that AA decreased the expression of these transcription factors involved in adipogenesis, we further analyzed the expression of the established adipocyte-specific marker genes, *lipoprotein lipase* (*Lpl*), *glucose transporter 4* (*Glut4*), and *Leptin* by RT-qPCR as described in Figure 6A–D show downregulated expression of *Lpl*, *Glut4*, and *Leptin*. Taken together, these results suggest that AA added during the differentiation phase may attenuate the activation of the IP receptor by PGI_2_ and prevent the progression of the early phase of adipogenesis triggered by *C/EBPβ* and *C/EBPδ* increased otherwise.

### 3.4. Effects of AA Coexistent with Pro-Adipogenic PGs during the Differentiation Phase of 3T3-L1 Cells on MDI-Induced Adipogenesis

**Figure 7:**
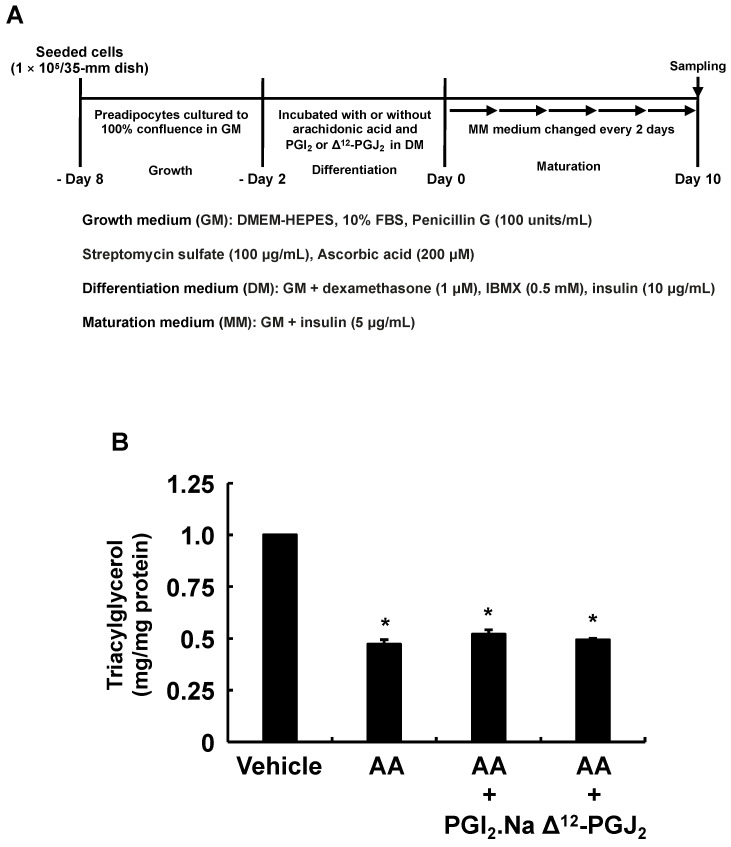
Effects of PGI_2_ or Δ^12^-PGJ_2_ on 3T3-L1 cells incubated with AA during the differentiation phase of 3T3-L1 cells. (**A**) Experimental procedure. We seeded and incubated 3T3-L1 cells (1 × 10^5^/dish) in 35 mm dishes in 2 mL of GM until they reached 100% confluence. The cells were then incubated for 48 h with 2 mL of DM containing vehicle, AA (50 µM), and PGI_2_.Na (100 nM) or Δ^12^-PGJ_2_ (1 µM). The differentiation medium was replaced with 2 mL of fresh MM every 2 days thereafter. Intracellular TAG levels were analyzed in terminally differentiated mature adipocytes on day 10. (**B**) Intracellular TAG levels in cultured adipocytes. Data are shown as means ± SEM of n = 3 experiments for (**B**). * *p* < 0.05 vs. control (vehicle in DM) (Tukey-Kramer tests). GM, growth medium; DM, differentiation medium; MM, maturation medium; AA, arachidonic acid; PGI_2_.Na, prostaglandin I_2_.Na; Δ^12^-PGJ_2_, Δ^12^-prostaglandin J_2_; TAG, triacylglycerol; SEM, standard error of the mean.

Since the production of PGI_2_ during the differentiation phase was reduced by the addition of AA (Figure 2E), we examined whether the inhibition of MDI-induced adipogenesis during the maturation phase by AA is alleviated by the coexistence of PGI_2_ with AA during only the differentiation phase (Figure 7A). Although addition of PGI_2_ during the differentiation phase promoted MDI-induced adipogenesis during the maturation phase (Figure 3B), the addition of PGI_2_ with AA during the differentiation phase was not able to rescue AA-stimulated inhibition of adipogenesis (Figure 7B). In addition, although the production of PPARγ activator, Δ^12^-PGJ_2_, during the differentiation phase was not changed by the addition of AA (Figure 2D), we also evaluated whether pro-adipogenic PGs other than PGI_2_ influence the AA-elicited inhibition of MDI-induced adipogenesis during the maturation phase (Figure 7A). As the case of PGI_2_, pro-adipogenic Δ^12^-PGJ_2_ (Figure 3B) was not able to rescue AA-stimulated inhibition of adipogenesis when Δ^12^-PGJ_2_ coexisted with AA during the differentiation phase (Figure 7B). Taken together, these results indicate that pro-adipogenic PGs production during the differentiation phase does not influence the suppression of MDI-induced adipogenesis during the maturation phase by adding AA during the differentiation phase.

## 4. Discussion

**Figure 8:**
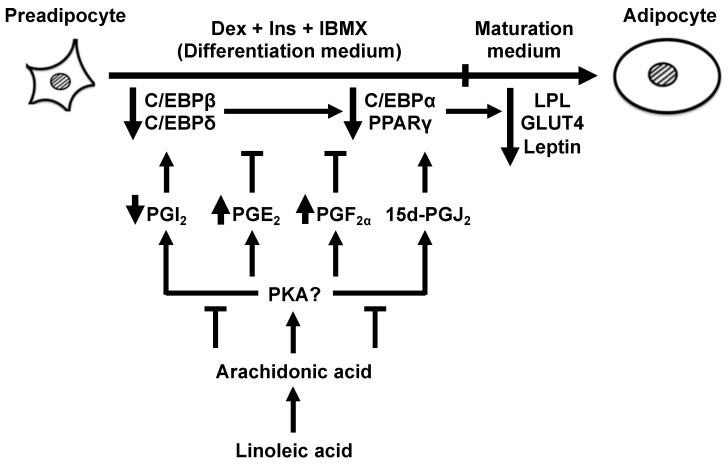
Schematic representation of arachidonic acid function during the differentiation phase of 3T3-L1 cells and subsequent effects on adipogenesis induced by 3-isobutyl-1-methylxanthine, dexamethasone, and insulin. Increased PGE_2_ and PGF_2α_ production, whereas the reduced PGI_2_ production together with the suppression of PGI_2_ and Δ^12^-PGJ_2_ actions by arachidonic acid during the differentiation phase inhibited MDI-induced adipogenesis during the maturation phase. C/EBPα, CCAAT/enhancer-binding protein-α; C/EBPβ, CCAAT/enhancer-binding protein-β; C/EBPδ, CCAAT/enhancer-binding protein-δ; DEX, dexamethasone; Ins, insulin; IBMX, 3-isobutyl-1-methylxanthine; PPARγ, peroxisome proliferator-activated receptor-γ; LPL, lipoprotein lipase; GLUT4, glucose transporter 4; PG, prostaglandin.

Figure 8 summarizes the present findings. In line with previous findings reported by other groups [28,29,31], the present study confirmed that AA added during the differentiation phase of 3T3-L1 cells inhibited MDI-induced adipogenesis during the maturation phase and elicited anti-adipogenic PGE_2_ and PGF_2α_ production. The novel findings are as follows: (1) LA added during only the differentiation phase did not inhibit MDI-induced adipogenesis during the maturation phase, even though LA is a precursor of AA; (2) Δ^12^-PGJ_2_ production was not affected by AA added during the differentiation phase; (3) PGI_2_ production was decreased by AA added during the differentiation phase, which in turn suppressed the progression of the adipogenic program initiated by increased *C/Ebpβ* and *C/Ebpδ* expression; (4) the addition of PGI_2_ or Δ^12^-PGJ_2_ with AA during the differentiation phase did not influence the AA-induced inhibition of MDI-induced adipogenesis during the maturation phase. Taken together, in addition to the alteration of PGs production, the inhibitory effects of AA on the actions of pro-adipogenic PGs during the differentiation phase may play an important role in the suppression of MDI-induced adipogenesis during the maturation phase.

Ingested LA must be metabolized to AA to affect white adipocytes in vivo [28]. The present and other studies have shown that AA participates in the inhibition of adipogenesis [28,29,31]. However, whether LA affects adipocytes in the same way as AA remains unclear. Our findings indicated that LA, unlike AA, did not inhibit adipogenesis induced by MDI, even though it is a precursor of AA. Thus, the present findings indicate that a reduction in white adipose tissue mass associated with ingesting LA from a high-protein/low-carbohydrate diet is due to the action of AA, and not LA. Furthermore, the present study indicates that the anti-adipogenic effects of AA are fully exerted when adipocytes are exposed to AA during the differentiation phase.

When AA is added to 3T3-L1 cells during the differentiation phase without IBMX, production of PGE_2_ and PGF_2α_ was decreased, while that of PGI_2_ increases in the phase, promoting the accumulation of intracellular TAG levels after maturation [30]. In contrast, the present study found that AA added during the differentiation phase with IBMX suppressed the accumulation of intracellular TAG levels after maturation. We also found increased PGE_2_ and PGF_2α_, and decreased PGI_2_ production in the differentiation phase. The only difference between the previous and the present studies is the presence or absence of IBMX added during the differentiation phase. Since IBMX is a cAMP-elevating agent, the difference in the types of PGs produced in the previous and current studies should be produced by cAMP-activated protein kinase A (PKA) or exchange protein directly activated by cAMP (Epac). Activation of both Epac and PKA during the differentiation phase contributes to MDI-induced adipogenesis [36,37], whereas activation of only PKA suppresses it [38]. Since MDI-induced adipogenesis was suppressed in the present study, only PKA may have been activated by adding IBMX in the presence of AA. In fact, the coexistence of H89, an inhibitor of PKA, with AA during the differentiation phase is reported to suppress the anti-adipogenic effects of AA [28]. Thus, during the differentiation phase, the change in PG production by adding AA depends on the presence of IBMX, suggesting the effect of PKA activation.

Since reduced PGI_2_ production leads to decreased *C/Ebpβ* and *C/Ebpδ* expression [15], we speculated that not only the rise in both anti-adipogenic PGs, PGE_2_, and PGF_2α_, but also the reduction in PGI_2_ production, by AA added during the differentiation phase was also responsible for the suppression of MDI-induced adipogenesis during the maturation phase. However, the inhibition of MDI-induced adipogenesis by AA during the maturation phase was not alleviated by the coexistence of PGI_2_ with AA during the differentiation phase. Based on these results, we also examined other pro-adipogenic PG, Δ^12^-PGJ_2_, and found similar results. These results proposed that the property of AA to nullify the effects of adipogenic PGs is the most significant factor leading to anti-adipogenic effects. Therefore, we speculate that the decreased *Pparγ* expression observed in the present study was primarily caused by the reduced PPARγ transcriptional activity rather than the reduced PGI_2_ production induced by AA. The reason supporting the speculation that decreased PPARγ transcriptional activity leads to decreased *Pparγ* expression is that PPARγ has an auto-loop mechanism with C/EBPα, and their transcriptional activity and expression levels correlate with each other [10]. In fact, this mechanism may be at work as well since a reduction in both *Pparγ* and *C/Ebpα* expression was observed in the present study. Reduced PPARγ transcriptional activity is induced by its phosphorylation through MAPK activation, and PGF_2α_ triggers this mechanism [39]. The observed increase in PGF_2α_ production induced by AA added during the differentiation phase in the present study may cause the decreased *Pparγ* expression through this mechanism. Furthermore, PGE_2_ also contributes to PGF_2α_ production by increasing COX-2 expression [40]. Based on these results, the increased PGE_2_ production induced by AA added during the differentiation phase may suppress MDI-induced adipogenesis during the maturation phase through the same mechanism as PGF_2α_ described above. A morphological evaluation of the effect of the addition of AA to the 3T3-L1 cells during the differentiation phase on adipogenesis, such as the size of intracellular lipid droplets as well as the shape and size of adipocytes, could provide us with a better understanding of the molecular mechanism governing the anti-adipogenic function of AA during the early phase of adipocyte differentiation. Since the above action mechanisms of AA are our speculation, further study will be needed in the future.

## 5. Conclusions

The present findings indicate that differentiation of cultured 3T3-L1 pre-adipocytes to matured adipocytes is attenuated by the addition of AA, but not LA, during the differentiation phase. The anti-adipogenic effects of AA could be explained, at least partially, by alteration in PG synthesis during the differentiation phase. The addition of AA increased anti-adipogenic PGE_2_ and PGF_2α_, while decreasing or not affecting pro-adipogenic PGI_2_ and Δ^12^-PGJ_2_. The addition of AA also decreased the expression of the differentiation-initiating genes, *C/Ebpβ* and *C/Ebpδ*, which could, by cooperating with the alteration in PG synthesis, decrease the expression of the following master-regulator genes, *Pparγ* and *C/Ebpα*, and finally the adipocyte-specific marker genes, *Lpl*, *Glut4*, and *Leptin*. AA should affect the very initiating steps in the differentiation as confirmed by the results that coexisting PGI_2_ or Δ^12^-PGJ_2_ did not cancel the AA-induced suppression of adipogenesis. PG synthesis is regulated by PKA: the presence of cAMP-increasing IBMX increases anti-adipogenic PGs, while its absence increases the production of pro-adipogenic PGs [30]. Therefore, controlling PKA activity by feeding, for example, the balance of carbohydrates and proteins in the diet, could be a determining factor for the effects of AA on the development of adipose tissue.

## Figures and Tables

**Table 1:** Forward (Fw) and reverse (Rv) primers for target genes.

Target Genes	Primers (5→3′)	Length (bp)	Tm (°C)	Product Length (bp)
*C/Ebpβ*	Fw: CGGGTTTCGGGACTTGAT	18	56.97	93
	Rv: GCCCGGCTGACAGTTACAC	19	61.03	
*C/Ebpδ*	Fw: GACTCCTGCCATGTACGACG	20	60.53	118
	Rv: GTTGAAGAGGTCGGCGAAGA	20	60.04	
*Pparγ*	Fw: CTTCGCTGATGCACTGCCTAT	21	60.81	216
	Rv: GGGTCAGCTCTTGTGAATGGA	21	60.00	
*C/Ebpα*	Fw: GCCAAGAAGTCGGTGGACA	19	59.93	110
	Rv: GTCTCCACGTTGCGTTGTTT	20	59.62	
*Lpl*	Fw: TTGCAGAGAGAGGACTCGGA	20	59.96	125
	Rv: GGAGTTGCACCTGTATGCCT	20	60.04	
*Glut4*	Fw: GGATTCCATCCCACAAGGCA	20	60.03	158
	Rv: CCAACACGGCCAAGACATTG	20	60.04	
*Leptin*	Fw: TTTCACACACGCAGTCGGTA	20	59.90	149
	Rv: CACATTTTGGGAAGGCAGGC	20	60.04	
*β-Actin*	Fw: GCGGGCGACGATGCT	15	59.84	197
	Rv: TGCCAGATCTTCTCCATGTCG	21	59.86	

## Data Availability

Not applicable.
